# GW2 Functions as an E3 Ubiquitin Ligase for Rice Expansin-Like 1

**DOI:** 10.3390/ijms19071904

**Published:** 2018-06-28

**Authors:** Beom Seok Choi, Yeon Jeong Kim, Kesavan Markkandan, Yeon Jong Koo, Jong Tae Song, Hak Soo Seo

**Affiliations:** 1 Department of Plant Science, Research Institute of Agriculture and Life Sciences, Seoul National University, Seoul 151-921, Korea; choebs@snu.ac.kr (B.S.C.); in12th79@seoul.ac.kr (Y.J.K.); kesavan@snu.ac.kr (K.M.); 2 Department of Biological Chemistry, Chonnam National University, Gwangju 61186, Korea; yeonjong@chonnam.ac.kr; 3 School of Applied Biosciences, Kyungpook National University, Daegu 41566, Korea; jtsong68@knu.ac.kr; 4 Plant Genomics and Breeding Institute, Seoul National University, Seoul 151-921, Korea; 5 Bio-MAX Institute, Seoul National University, Seoul 151-818, Korea

**Keywords:** chalky endosperm, E3 ubiquitin ligase, EXPLA1, GW2, rice, seed size, ubiquitination

## Abstract

Seed size is one of the most important traits determining the yield of cereal crops. Many studies have been performed to uncover the mechanism of seed development. However, much remains to be understood, especially at the molecular level, although several genes involved in seed size have been identified. Here, we show that rice Grain Width 2 (GW2), a RING-type E3 ubiquitin ligase, can control seed development by catalyzing the ubiquitination of expansin-like 1 (EXPLA1), a cell wall-loosening protein that increases cell growth. Microscopic examination revealed that a *GW2* mutant had a chalky endosperm due to the presence of loosely packed, spherical starch granules, although the grain shape was normal. Yeast two-hybrid and in vitro pull-down assays showed a strong interaction between GW2 and EXPLA1. In vitro ubiquitination analysis demonstrated that EXPLA1 was ubiquitinated by GW2 at lysine 279 (K279). GW2 and EXPLA1 colocalized to the nucleus when expressed simultaneously. These results suggest that GW2 negatively regulates seed size by targeting EXPLA1 for degradation through its E3 ubiquitin ligase activity.

## 1. Introduction

Rice (*Oryza sativa* L.) yield is regulated by various factors, including tiller and panicle numbers and seed size. Among these factors, seed size is a major yield-determining trait [1,2,3]. The grain weight of rice is directly related to the seed size and thickness, which are in turn controlled by the hull. To understand the regulatory mechanisms of seed development, many groups have worked for over two decades to identify genes involved in the control of grain size. This has resulted in the identification of many QTLs (quantitative trait loci), most of which correspond to genes that control grain length and width [4]. One of these is the *GW2* gene, which encodes an E3 ubiquitin ligase containing a RING finger motif and possessing autoubiquitination activity [3]. A one-base-deleted *GW2* mutant produces larger rice seed, and GW2 negatively regulates cell division [3]. GW2 homologues were also identified in wheat (*Triticum aestivum* L.) and maize (*Zea mays*). The *GW2* gene *TaGW2* is also involved in the determination of kernel size and maturity [5]. Two maize *GW2* genes, namely, *ZmGW2-CHR4* and *ZmGW2-CHR5*, influence kernel weight and kernel width, respectively [6].

Plant growth is mediated by sequential increases in cell size and number. Increases in cell size occur via cell expansion, an important mechanism involved in the development of plant tissues and organs that depends on the action of enzymes such as expansin and xyloglucan endotransglucosylase/hydrolase (XTH) [7,8,9]. Expansins, small cell wall proteins composed of 225 to 300 amino acid residues [10], break the hydrogen bonds linking cellulose and hemicellulose in the plant cell wall [11] and are grouped into four families [12]: α-expansin or expansin A (EXPA), β-expansin or expansin B (EXPB), expansin-like A (EXPLA), and expansin-like B (EXPLB). Expansins affect almost all plant growth stages and influence biotic and abiotic stress relationships [13]. In rice, four expansins, EXPA1, EXPA4, EXPA8, and EXPA17, have been shown to influence coleoptile growth, internode development, plant height, leaf number and size, root development, flowering time, and seed set [13].

Ubiquitin, a small polypeptide composed of 76 amino acids, undergoes covalent attachment to target proteins via a process called ubiquitination [14], which requires the sequential action of three enzymes, E1, E2, and E3. E1 is a ubiquitin-activating enzyme that is covalently modified by ubiquitin. E2 is a ubiquitin-conjugating enzyme that is conjugated to activated ubiquitin. E3 is a specific ubiquitin ligase that interacts with ubiquitin-conjugated E2 enzyme. E3 has autoubiquitination and substrate ubiquitination activities [15,16]. Ubiquitination is involved in the regulation of localization, stability, and activity of the modified proteins. Target proteins can undergo monoubiquitination or polyubiquitination. Polyubiquitinated target proteins, but not monoubiquitinated proteins, are degraded by the 26S proteasome complex. Monoubiquitination affects the localization or function of the modified protein [16]. Ubiquitination is emerging as a key process in the regulation of the seed size of crop plants. Although recent progress has been made towards the cloning and characterization of seed size-related QTLs and genes, the mechanisms determining the seed size are still unclear. The characterization of GW2 function through the identification of its substrates could help provide information on the mechanisms that determine seed size.

Here, we elucidated a possible mechanism of GW2-mediated seed size control. GW2 directly interacts with and ubiquitinates expansin-like 1 (EXPLA1), which can inactivate it. Our results suggest that GW2 negatively regulates cell division and expansion by inducing EXPLA1 ubiquitination through its E3 ubiquitin ligase activity.

## 2. Results

### 2.1. The Seeds of GW2 Mutants Have a Chalky Endosperm

Grain size is tightly regulated by the spikelet hull [17]. The spikelet hull of grain with the WY3 allele of *GW2* is markedly wider and longer than that of the wild-type FAZ1 [2]. With this in mind, we chose the natural *gw2* mutant, Oochikara, which is similar to WY3, for this study. Norin is a parental cultivar of Oochikara. The *GW2* allele of Oochikara has an identical sequence to that of the WY3-*GW2* allele. Nucleotide sequence analysis of the Oochikara allele showed a one-base-pair deletion at A314, as in the WY3-*GW2* allele [2], resulting in a premature stop codon and the production of a truncated protein.

**Figure 1:**
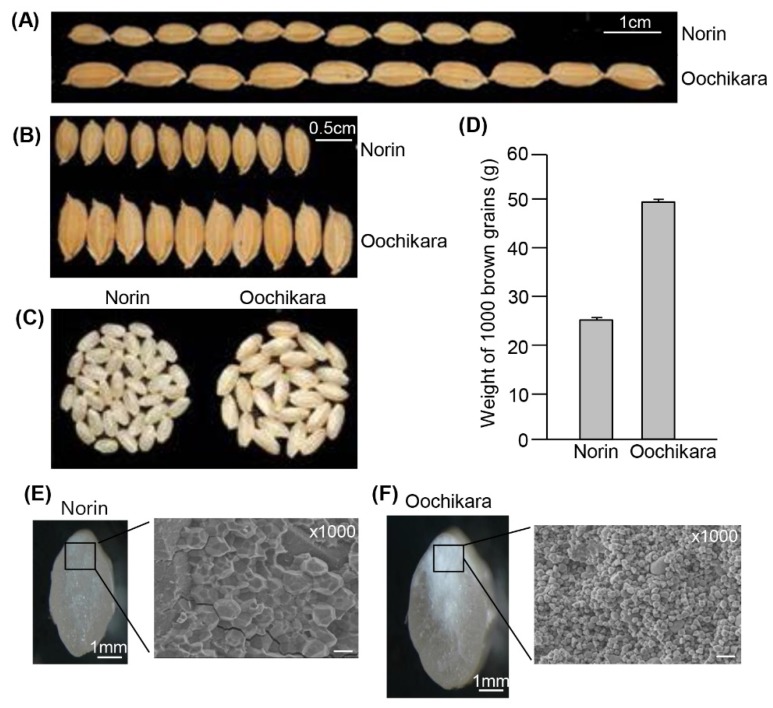
Phenotypic analysis of wild-type and *gw2* mutant grains. (**A**–**C**) Grain phenotypes of Norin and Oochikara. Grain length (**A**) and width (**B**) of ten seeds of Norin and Oochikara. Brown grains from which husks were removed (**C**); (**D**) Weight of 1000 brown grains of Norin and Oochikara. Error bars indicate standard deviations (*n* = 3); Palea and lemma of the grains of Norin (**E**) and Oochikara (**F**) were removed. The seeds were hand-sectioned with a razor blade transversely and then analyzed by SEM. Scale bar, 10 μm.

Phenotypic analysis revealed that Oochikara seeds did not differ substantially from wild-type seeds in visible features, except for the substantial difference in size and weight (Figure 1A–D), as observed previously for WY3 [2]. Thus, we further examined the grain phenotype of Oochikara using cross-sectioned seeds and found that Oochikara seeds had a chalky and floury endosperm. Scanning electron microscopy analysis of transversely sectioned endosperm revealed that the Oochikara had spherical starch granules that were loosely packed together (Figure 1F), unlike the irregular polyhedral starch granules of the wild-type seeds, which were densely packed (Figure 1E).

### 2.2. EXPLA1 Specifically Interacts with GW2

Because GW2 is a RING-domain protein that has autoubiquitination activity, we hypothesized that it would function as an E3 ubiquitin ligase for a target protein involved in GW2-mediated seed development. Therefore, to identify the proteins that interact with GW2, we performed a yeast two-hybrid screen using the GW2-Nter (GW2-N terminus, 1–205 amino acids) as the bait and a cDNA library constructed from developing rice seeds at 15 DAF as the prey. Because of the transcription activation activity of the GW2-C terminus (in particular residues 205 to 260), the GW2-N terminus was used as the bait. We screened approximately half a million yeast colonies and found that 28 candidates had a strong affinity for GW2-Nter (Table 1). Plasmid extraction, DNA sequencing, and database searches revealed that 13 out of 19 clones contained cDNA for polyubiquitin, and two corresponded to ubiquitin- or S10/S20 domain-containing ribosomal proteins. The remaining four clones corresponded to ubiquitin-conjugating enzyme (Os01g60410), polyphenol oxidase (Os04g53300), glycosyl hydrolase (Os06g46284), and EXPLA1 (Os03g04020) (Table 1).

**Figure 2:**
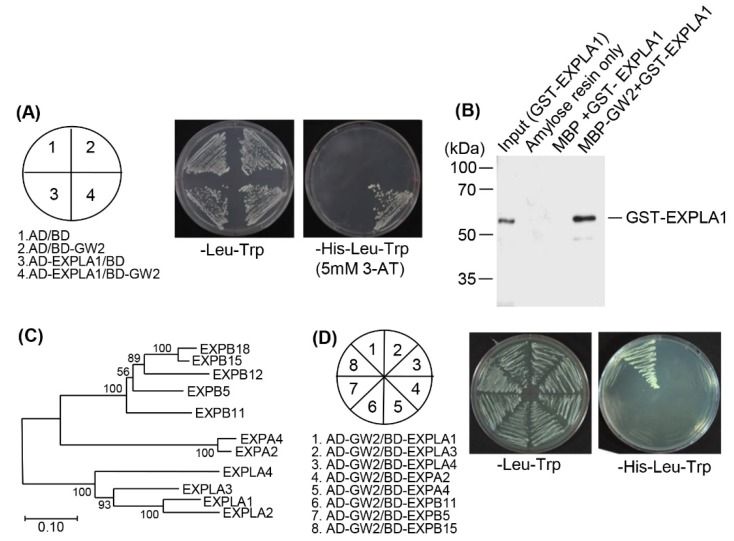
Grain Width 2 (GW2) and expansin-like 1 (EXPLA1) specifically interact in vivo and in vitro. (**A**) *GW2* and *EXPLA1* cDNAs were fused to sequences encoding the Gal4 activation domain (AD) or the Gal4 DNA-binding domain (BD) in pGAD424 and pGBT8, respectively. GW2 and EXPLA1 constructs were co-transformed into the yeast strain AH109. The transformants were plated onto minimal medium Leu/-Trp or Leu/-Trp/-His, including 5 mM 3-AT, and incubated for 4 days; (**B**) In vitro pull-down assays. MBP, MBP–GW2, and GST–EXPLA1 were overexpressed in *Escherichia coli* and purified using amylose (MBP) or glutathione (GST) resins, and then EXPLA1 was pulled down with GW2. GST–EXPLA1 bound to MBP–GW2 was detected by western blotting with an anti-GST antibody; (**C**) Phylogenic analysis between EXPLA1 and its homologs. The amino acid sequences of EXPLA1 and its homologs were aligned by CLUSTAL W, and the phylogenetic tree was constructed by MEGA 7.0 using the neighbor-joining method. Bootstrap values are shown for each node; (**D**) Examination of the interaction between GW2 and EXPLA1 and between GW2 and EXPLA1 homologs by yeast two-hybrid analysis. Full-length cDNAs encoding EXPLA1 homologs were fused to sequences encoding the Gal4 BD in pGBT8. The constructs for GW2 and each of the EXPLA1 homologs were co-transformed into the yeast strain AH109. After selection of the transformants on the minimal medium Leu/-Trp, the interaction between GW2 and each EXPLA1 homolog was examined on the minimal medium Leu/-Trp/-His.

Expansins participate in cell wall loosening, which results in cell expansion [11]. We thus chose EXPLA1 for further experiments. We re-examined the interaction between GW2 and EXPLA1 by the yeast two-hybrid assay. *GW2* and *EXPLA1* cDNAs were inserted into yeast expression vectors, and the constructs were introduced into the yeast strain AH109. As expected, GW2 strongly interacted with EXPLA1 (Figure 2A)*.* An in vitro pull-down assay confirmed this interaction. Two recombinant plasmids were constructed: MBP-tagged GW2 and GST-tagged EXPLA1. The recombinant proteins MBP–GW2 and GST–EXPLA1 were overexpressed in *Escherichia coli*, purified on affinity columns, and then GST–EXPLA1 was pull-downed with MBP or MBP–GW2. Consistent with the results of the yeast two-hybrid assay, we observed a strong interaction between GW2 and EXPLA1 (Figure 2B).

We also tested for a possible interaction between GW2 and EXPLA1 homologs. To do this, we first searched for EXPLA1 homologs using a phylogenic tree (Figure 2C). On the basis of the data obtained, we chose seven proteins, EXPLA3, EXPLA4, EXPA2, EXPA4, EXPB5, EXPB11, and EXPB15, and then performed a yeast two-hybrid screening using their full-length cDNAs. EXPLA1 was used as a positive control. As a result, we found that none of the homologs interacted with GW2 (Figure 2D), indicating that GW2 interacts specifically with EXPLA1.

### 2.3. GW2 and EXPLA1 Colocalize to the Nucleus

**Figure 3:**
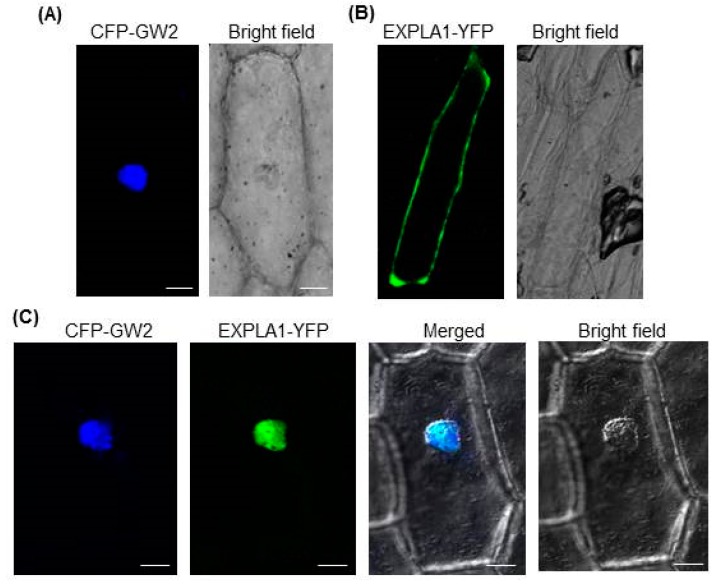
GW2 and EXPLA1 colocalize in the nucleus. CFP–GW2 and EXPLA1–YFP were transiently expressed in onion epidermal cells. Scale bar, 50 μm. CFP–GW2 (**A**) and EXPLA1–YFP (**B**) were distributed in the nucleus and cell wall, respectively, when expressed independently; (**C**) CFP–GW2 and EXPLA1–YFP were localized in the nucleus. Scale bar, 50 μm.

Expansins are enzymes located in the cell wall [18] that break hydrogen bonds linking cellulose and hemicellulose [11]. Our results showed a strong interaction between GW2 and EXPLA1 (Figure 2A,B), suggesting that they localize to the same cellular compartment. To test this possibility, we investigated the subcellular localizations of GW2 and EXPLA1 using onion epidermal cells. The results showed that when they were expressed individually, CFP–GW2 was localized to the nucleus (Figure 3A) and EXPLA1–YFP was localized to the cell wall (Figure 3B). However, when they were expressed together, they both localized to the nucleus (Figure 3C).

### 2.4. EXPLA1 Is Ubiquitinated by GW2

**Figure 4:**
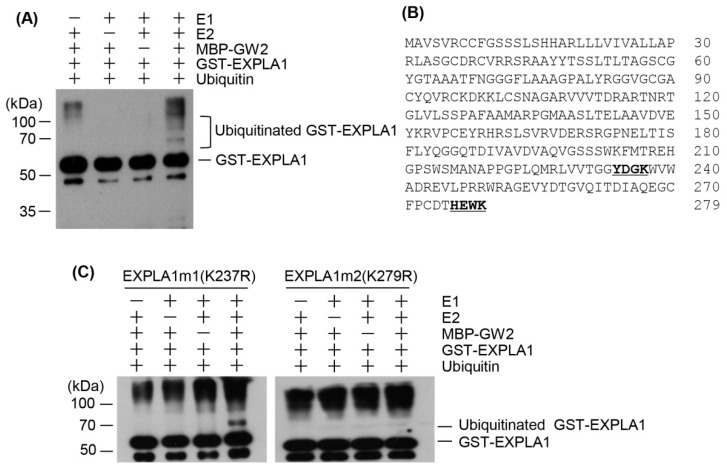
EXPLA1 is ubiquitinated by GW2 in vitro. (**A**) The ubiquitination reactions were performed in the presence or absence of rabbit E1, UbcH5b (E2), MBP–GW2 (E3), His_6_-ubiquitin, and GST–EXPLA1. Ubiquitinated EXPLA1 was detected by western blotting using an anti-GST antibody; (**B**) Deduced amino acid sequences of the EXPLA1 protein. Two putative ubiquitination sites are underlined in bold; (**C**) In vitro identification of the ubiquitination site on EXPLA1. The ubiquitination reaction mixture contained E1, E2, MBP–GW2 (E3), and His_6_-ubiquitin without (−) or with (+) a substrate protein. The mutant proteins, EXPLA1m1 and EXPLA1m2, have amino acid substitutions at residues that are predicted to be ubiquitin conjugation sites in EXPLA1, as indicated. After the reaction, ubiquitinated EXPLA1 protein was detected by western blotting with an anti-GST antibody.

The direct interaction between GW2 and EXPLA1 and their colocalization suggested that GW2 may act as an E3 ubiquitin ligase for EXPLA1. To investigate this, we expressed recombinant MBP–GW2 and GST–EXPLA1 proteins in *E. coli* and purified them using affinity columns. In vitro ubiquitination assays showed that GST–EXPLA1 was ubiquitinated by GW2 in an E1- and E2- dependent manner (Figure 4A), indicating that GW2 has E3 ubiquitin ligase activity towards EXPLA1. Next, we attempted to identify the ubiquitination site on EXPLA1. The predicted amino acid sequence of EXPLA1 contains two putative ubiquitination sites located at lysines 237 (K237) and 279 (K279) (Figure 4B). Therefore, two mutated EXPLA1 proteins, GST–EXPLA1m1 (K237R) and GST–EXPLA1m2 (K279R), were generated and purified on glutathione columns after overexpression in *E. coli*. In vitro ubiquitination assays showed that GST–EXPLA1m2 was not ubiquitinated, whereas GST–EXPLA1m1 was clearly ubiquitinated (Figure 4C).

### 2.5. GW2 Is Highly Expressed in Panicle and Anther

We examined GW2 expression using GUS and GFP reporters. To do this, 3.0 kb GW2 promoter was cloned in the pMDC163 or pMDC107 gateway vector [19]. The resulting constructs, *GW2pro–GUS* and *GW2pro–GFP* (Supplementary Figure S1A), were introduced into rice, and then histochemical and fluorescence assays were performed after flowering. The GUS assay showed that GW2 was strongly expressed in young panicle and anther (Supplementary Figure S1B), as observed previously. GFP signals were also strongly detected in young panicle and anther (Supplementary Figure S1C). However, the GUS and GFP levels were low in the endosperm (Supplementary Figure S1B,C).

## 3. Discussion

Seed size is one of the main factors determining the economic value of seeds, and, thus, much effort has been expended to increase seed size. However, seed size-related genes remain poorly characterized, and the mechanisms that control seed size remain largely unknown. Thus, new studies are required to identify new proteins involved in the determination of rice grain size. Here, we functionally characterized GW2 as a seed size-related gene through the identification of one of its substrates.

Various genes participate in the control of grain width and length by changing the cell size in the spikelet hull [1,3,19,20,21,22]. The natural *gw2* mutant Oochikara had increased seed width, length, and weight (Figure 1A–D). In addition, it had floury endosperm caused by loosely packed small and spherical starch granules (Figure 1F), which suggests that GW2 loss changes the stability or activity of target proteins involved in endosperm development, including those involved in the formation of the starch granules and protein bodies that lead to a floury endosperm.

GW2 contains a RING finger motif and has autoubiquitination activity [2], suggesting that it may ubiquitinate proteins involved in the regulation of cell size in the spikelet hull. Yeast two-hybrid analysis showed that GW2 interacted predominantly with polyubiquitin proteins (Table 1), which is consistent with previous reports showing that qSW5/GW5 interacts with polyubiquitin [20]. This suggests that qSW5/GW5 and GW2 may function together in the ubiquitin-dependent proteasome pathway. Nevertheless, the functional relationship between the two proteins remains to be investigated. Among the proteins that interacted with GW2 (Table 1), we identified glycosyl hydrolase (Os06g46284) and EXPLA1 (Os03g04020), which can function in cell wall extension [23,24,25]. However, the mechanism by which EXPLA1 participates in the regulation of seed size and weight has not been identified, although it can function as a cell wall-loosening protein that regulates cell wall enlargement in growing cells. Recently, gene expression analyses, including transcriptome analysis and temporal and spatial expression profiling, suggested that expansin genes are involved in determining grain size and yield [26,27]. More recently, it was reported that transgenic Arabidopsis overexpressing bEXP1, a sweet potato expansin, produces larger seeds [28]. Notably, our results showed that EXPLA1 was ubiquitinated by GW2 through direct interaction in vitro (Figure 2A,B and Figure 4A), and GW2 did not interact with the tested expansins or expansin-like proteins other than EXPLA1 (Figure 2D), indicating that it acts as a specific E3 ubiquitin ligase for EXPLA1. This suggests that EXPLA1 can participate in GW2-mediated seed development, although there is no direct evidence that EXPLA1 regulates seed size. All of these results need to be confirmed in vivo. In addition, it is still possible that EXPLA2 also interacts with GW2 because EXPLA2 was classified into the same group as EXPLA1 (Figure 2C).

EXPLA1 ubiquitination by GW2 can only occur if the two proteins are present within the same cell compartment. Transient expression assays using onion epidermal cells showed that GW2 and EXPLA1 were present in the nucleus and cell wall, respectively, when expressed individually (Figure 2A,B). Previous studies showed that α-expansins [29] and β-expansins [30] are bound to the cell wall, suggesting that expansins, including EXPLA1, are localized to the cell wall. Both GW2 and EXPLA1 localized to the nucleus when expressed simultaneously (Figure 3C). A previous study reported that GW2 localizes to the cytoplasm [2]. However, under our experimental conditions, we always found that GW2 was localized to the nucleus when expressed alone or together with EXPLA1, although a very weak GW2 signal was sometimes detected in the cytoplasm. Currently, we do not know the reason for the difference between our results and those of the previous study. Nonetheless, our results suggest that EXPLA1 is ubiquitinated as a result of GW2 E3 ligase activity in the nucleus. However, we do not know under which physiological conditions and by which mechanism GW2 interacts with EXPLA1. We suggest two possibilities. First, if EXPLA1 does not move from the cell wall to the nucleus, it may directly enter the nucleus after its synthesis in the endoplasmic reticulum. In this case, EXPLA1 may not have any function at all because it may be ubiquitinated by GW2 activity through direct interaction. Alternatively, cell wall-bound EXPLA1 may be transported to the nucleus by another protein after it has fulfilled its function and then ubiquitinated by GW2 activity in the nucleus. EXPLA1 can be degraded via the 26S proteasome complex after ubiquitination by GW2. To determine the ubiquitination site in EXPLA1, we produced two mutant proteins, EXPLA1m1 (K237R) and EXPLA1m2 (K279R), and examined their ubiquitination in vitro. EXPLA1m1, but not EXPLA1m2, was ubiquitinated (Figure 4C), indicating that K279 is a ubiquitination site, at least in vitro. Further investigation with transgenic rice overexpressing EXPLA1m2 will be required to confirm the link between seed size and EXPLA1 ubiquitination.

Investigation of GW2 expression during flower and seed development using transgenic rice plants transformed with *GW2_pro_-Gus* or *GW2_pro_-GFP* showed that GW2 was highly expressed in the anther and husk. GW2 expression was quite weak in the endosperm, although GUS and GFP signals were detected in some parts of the endosperm (Supplementary Figure S1B,C). A previous study reported constitutive expression of *GW2* in the endosperm [2]. It is possible that we were unable to detect weak GUS or GFP signals dispersed in the endosperm by microscopy, whereas low levels of mRNA were amplified by RT-PCR in the previous study. Previous data and our data indicate that *GW2* is mainly expressed at an early stage of seed development, which allows it to modulate anther and husk development after heading.

*GW5*/*qSW5* and *HGW* genes identified by map-based cloning encode a polyubiquitin-interacting nuclear protein and a novel plant-specific ubiquitin domain protein, respectively [20,31], which strongly suggests that GW5/qSW5, HGW, and GW2 function in the same ubiquitination pathway for the regulation of rice grain size and weight, although they may function with different target proteins. Very recently, six genes *DEP1*, *GS7*, *GS3*, *GW8*, *GL7*, and *GS*, but not *GW2*, were reported to be strongly associated with grain size traits [32], suggesting that *GW2* acts in a different pathway from these genes.

In summary, we conclude that GW2 has E3 ligase activity towards EXPLA1. To date, no data on interaction partners for the seed size-regulating protein GW2 have been published. Hence, this is the first report that GW2 function may be mediated through a target protein in rice. We are now producing transgenic Norin and Oochikara plants overexpressing EXPLA1 and EXPLA1m2. Further analysis of the transgenic plants will provide insights into how EXPLA1 and the ubiquitination system participate in GW2-mediated regulation of seed size, weight, and phenotype in rice.

## 4. Materials and Methods

### 4.1. Bacteria, Plant Materials, and Growth Conditions

*Escherichia coli* strains DH5α, DH10B, and Top10 (Invitrogen, Waltham, MA, USA) were used for cloning and plasmid preparation. BL21/DE3 pLysS (Invitrogen) was used to express recombinant proteins. Wild-type, mutant, and transgenic rice were grown at 26 °C under long-day conditions (16 h light/8 h dark) in a greenhouse or field. The japonica natural *gw2* mutant Oochikara (Accession No. 54075) was obtained from the National Institute of Agrobiological Sciences (NIAS), Tsukuba, Japan, and the wild-type Norin 22 was kindly provided by Dr. Hee Jong Koh, Seoul National University.

### 4.2. Genotyping of Natural gw2 Mutant Oochikara

The field-grown wild type (Norin) and natural *gw2* mutant (Oochikara) were subjected to polymerase chain reaction (PCR) genotyping. Genomic DNA was isolated from 30-day-old leaves of wild-type and *gw2* mutant plants according to the CTAB extraction method [17]. Genomic DNA encoding the GW2 protein was cloned by PCR with gene-specific primers, and nucleotide sequences were identified by an ABI 3730 automated DNA sequencer. The primers used in this study are listed in Supplementary Table S1.

### 4.3. Yeast Two-Hybrid Screening

*GW2* cDNA encoding the N-terminus (*GW2-Nter*) from 1 to 205 amino acids was amplified by PCR with gene-specific primers and then introduced into the pGBKT7 vector, containing a binding domain (BD) (Clontech, Mountain view, CA, USA). A cDNA library was constructed using seeds harvested 10 days after flowering (DAF). Interacting partners were isolated using the BD Matchmaker Library Construction and Screening Kit (Clontech, Mountain view, CA, USA), according to the manufacturer’s instructions. Yeast strain Y187 was transformed with *BD-GW2-Nter*, and yeast strain AH109 was transformed with an activating domain (AD) fusion library, and then the transformed Y187 cells were mated with the transformed yeast strain AH109. The cells were grown on media plates lacking histidine, leucine, and tryptophan. After screening by subculture in the same media three times, the candidate clones were used for further analysis.

### 4.4. Yeast Two-Hybrid Assay

To confirm the interaction between GW2 and EXPLA1, full-length *GW2* and *EXPLA1* cDNAs were cloned into pGAD424 and pGBT8 (Clontech), respectively. The recombinant plasmids, *AD-GW2* and *BD-EXPLA1*, were introduced into yeast strain AH109 using the lithium acetate method, and then transformed cells with *AD-GW2* and *BD-EXPLA1* constructs were selected by growing them on minimal medium (−Leu/−Trp). The growth of the selected cells was examined on minimal medium (−Leu/−Trp/−His/5 mM 3-AT (3-amino-1, 2, 4-triazole)) to confirm the interaction between GW2 and EXPLA1.

The interaction between GW2 and EXPLA1 homologs was also examined by yeast two-hybrid. Full-length cDNAs encoding *EXPLA3*, *EXPLA4*, *EXPA2*, *EXPA4*, *EXPB11*, *EXPB5*, and *EXPB15* were cloned into pGBT8 (Clontech). The recombinant plasmids expressing *AD-GW2* and *BD-EXPLA1* homologs were transformed into the yeast strain AH109, and further analysis was performed as described above.

### 4.5. Construction of Recombinant Plasmids

To produce maltose-binding protein (MBP)–GW2 and glutathione S-transferase (GST)–EXPLA1, the cDNA sequences encoding full-length *GW2* and *EXPLA1* were amplified by PCR with gene-specific primers and cloned in pGEX4T-1 (Amersham Biosciences) and pMALc2 (New England Biolabs, Ipswich, MA, USA) vectors, respectively. To produce the EXPLA1 mutant proteins GST–EXPLA1m1 (K237R) and GST–EXPLA1m2 (K279R), GST–EXPLA1 was subjected to site-directed mutagenesis using overlapping primers. The codons for Lys 237 and Lys 279 in the EXPLA1 coding region were changed to Arg, and primers were designed accordingly. Recombinant plasmids were introduced into the *E. coli* strain BL21, and the recombinant proteins were expressed by treating the cells with 5 mM of isopropyl-*β*-d-thiogalactoside (IPTG). K237R and K279R indicate that lysines at positions 237 and 279 were mutated to arginines.

### 4.6. Purification of Recombinant Proteins

The recombinant proteins were purified according to the manufacturer’s instructions. MBP and MBP–GW2 were purified by resuspending and sonicating the recombinant cells expressing these proteins in a buffer containing 20 mM Tris-HCl (pH 7.4), 200 mM NaCl, 1 mM EDTA, 1% Triton X-100, 2 mM PMSF, and a proteinase inhibitor cocktail (Roche, Basel, Switzerland). Total protein extracts were loaded onto a column packed with amylose resin (New England Biolabs). After washing the column with buffer containing 20 mM Tris-HCl (pH 7.4), 200 mM NaCl, 1 mM EDTA and 1% Triton X-100, the recombinant proteins were eluted with 10 mM maltose. GST–EXPLA1, GST–EXPLA1m1, and GST–EXPLA1m2 were purified by resuspending and sonicating the cells expressing these proteins in buffer containing 50 mM Tris, 50 mM NaCl, 1 mM EDTA, 1% Triton X-100, 2 mM PMSF, and a proteinase inhibitor cocktail (Roche). Total protein extracts were loaded onto a column packed with glutathione resin (Pharmacia, Piscataway. NJ, USA). After washing the column with buffer containing 50 mM Tris, 50 mM NaCl, 1 mM EDTA and 1% Triton X-100, the recombinant proteins were eluted with 10 mM glutathione. The protein concentrations were determined by the Bradford assay (Bio-Rad, Hercules, CA, USA).

### 4.7. In Vitro Pull-Down Assays

In vitro binding of MBP–GW2 to GST–EXPLA1 was performed in reaction buffer containing 50 mM Tris-HCl (pH 7.5), 100 mM NaCl, 1% Triton X-100, 0.2% glycerol, and 0.5 mM *β*-mercaptoethanol. After addition of 2 µg MBP–GW2 and 2 µg GST–EXPLA1, the reaction mixtures were incubated at 25 °C for 2 h and then pull-downed with amylose resin. The resins were washed with buffer containing 50 mM Tris-HCl (pH 7.5), 100 mM NaCl, and 1% Triton X-100 six times and then eluted with 10 mM glutathione. The eluted proteins were separated by 10% sodium dodecyl sulfate (SDS)-polyacrylamide gel electrophoresis (PAGE), and GST–EXPLA1 was detected by immunoblotting with an anti-GST antibody (0.4 μgmL^−1^; Santa Cruz Biotechnology, Santa Cruz, CA, USA).

### 4.8. Subcellular Localization of GW2 and EXPLA1

The cellular localizations of GW2 and EXPLA1 were determined by generating the recombinant plasmids 35S-*CFP–GW2* and 35S-*EXPLA1–YFP*. The 5′-end of the *GW2* gene was fused with the *CFP* gene, and the 3′-end of the *EXPLA1* gene was fused with the YFP gene. The recombinant plasmids or combinations thereof were introduced into onion epidermal cells by particle bombardment. After incubation for 15 h in the dark, CFP and YFP fluorescence was analyzed by confocal microscopy.

### 4.9. In Vitro Ubiquitination Assay

In vitro ubiquitination reactions were carried out in a reaction buffer containing 50 mM Tris-HCl (pH 7.4), 5 mM MgCl_2_, 150 mM NaCl, 2 mM ATP, and 1 mM DTT. Each reaction mixture contained 50 ng E1 (Boston Bochem, Cambridge, MA, USA), 100 ng E2 (Boston Bochem), 5 µg His_6_-ubiquitin (Sigma, Saint Louis, MO, USA), 100 ng GST-EXPLA1, and 200 ng MBP-GW2. The reactions were performed at 30 °C for 3 h, and then the reaction mixtures were separated by 10% SDS-PAGE. Ubiquitinated GST–EXPLA1 was detected by immunoblotting with an anti-GST antibody. To identify the ubiquitination site in EXPLA1, GST–EXPLA1m1 or GST–EXPLA1m2 was added to the reaction mixtures instead of GST–EXPLA1. The reactions and subsequent steps were performed as described above.

### 4.10. Promoter Activity Analysis

A 3.0 kb GW2 promoter was amplified from genomic DNA by PCR with gene-specific primers, and the PCR product was cloned in the pMDC163 and pMDC107 gateway vectors [33]. The resulting *GW2_pro_–GUS* and *GW2_pro_–GFP* constructs were introduced into Agrobacterium EHA105 and then further transformed into the japonica cultivar Dongjinbyeo. After selection of transgenic plants, histochemical assays were carried out as described previously [19]. The specimens were photographed using the OLYMPUS SZX7 microscope system. GFP fluorescence of flowers and immature seeds of transgenic and non-transgenic plants was observed using a fluorescence microscope (LAS 4000 imager, GE Life Science, Chicago, IL, USA).

### 4.11. Microscopic Analysis of gw2 Mutant Seeds

The starch granules on the surface of endosperms of *gw2* mutant seeds were observed using scanning electron microscopy (SEM). Mature seeds of wild-type and *gw2* mutant were transversely sectioned with a razor blade, and the starch granules were examined as described previously [20]. The starch granule morphology of wild-type and *gw2* mutant seeds was also observed using powder extracted from wild-type and *gw2* mutant mature seeds, as described previously [20].

## Figures and Tables

**Table 1:** List of GW2-interacting proteins that were isolated by yeast two-hybrid screening.

Colony	Protein ID	Inserted Region	Full Length
1	Ubiquitin family protein, Os06g46770, (polyubiquitin)	378–534aa	(534aa)
2	Ubiquitin family protein, Os06g46770, (polyubiquitin)	204–534aa	(534aa)
5	Ubiquitin family protein, Os06g46770, (polyubiquitin)	258–534aa	(534aa)
8	Ubiquitin-conjugating enzyme, Os01g60410	1–149aa	(149aa)
9	Ubiquitin family protein, Os02g06640, (polyubiquitin)	28–121	(458aa)
11	Ubiquitin family protein, Os02g06640, (polyubiquitin)	316–458aa	(458aa)
12	Ubiquitin family protein, Os06g46770, (polyubiquitin)	456–534aa	(534aa)
13	Ubiquitin family protein, Os02g06640, (polyubiquitin)	279–458aa	(458aa)
14	Ubiquitin family protein, Os02g06640, (polyubiquitin)	355–458aa	(458aa)
15	Ubiquitin family protein, Os06g46770, (polyubiquitin)	454–534aa	(534aa)
16	Ubiquitin family protein, Os06g46770.3, (polyubiquitin)	302–458aa	(458aa)
18	Ubiquitin family protein, Os02g06640, (polyubiquitin)	259–458aa	(458aa)
20	Expansin precursor, Os03g04020, (EXPLA1)	154–280aa	(280aa)
21	Ubiquitin family protein, Os06g46770.3, (polyubiquitin)	354–458aa	(458aa)
22	Ubiquitin family protein, Os06g46770.3, (polyubiquitin)	160–458aa	(458aa)
24	Polyphenol oxidase, Os04g53300	467–571aa	(1713aa)
25	Glycosyl hydrolase, Os06g46284	760–886aa	(886aa)
26	Ubiquitin-40S ribosomal protein, Os01g22490	1–156aa	(156aa)
28	S10/S20 domain containing ribosomal protein, Os06g04290	50–129aa	(129aa)

## Supplementary Materials

Supplementary materials can be found at http://www.mdpi.com/1422-0067/19/7/1904/s1.
