# Supplementary material for: GW2 Functions as an E3 Ubiquitin Ligase for Rice Expansin-Like 1
Source: Int J Mol Sci. 2018 Jun 28;19(7):1904. doi: 10.3390/ijms19071904 (PMC6073362; doi:10.3390/ijms19071904)
Supplement: Supplementary file 1 [file ijms-19-01904-s001.pdf]

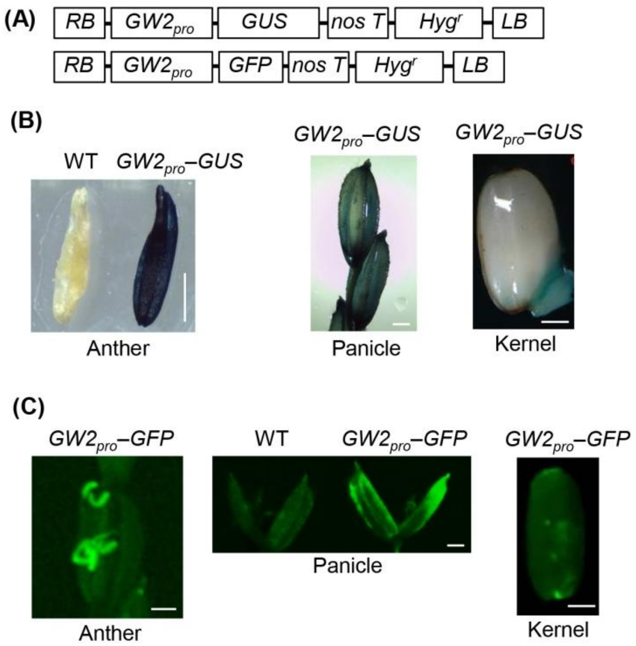

Supplementary Figure S1. GUS and GFP expression patterns directed by the GW2 promoter. (**A**) Binary vectors for GW2 promoter analysis, with the promoter region fused to the *GUS* or *GFP* reporter genes. RB, right border; $GW2_{pro}$, GW2 promoter; GUS, β-glucuronidase; GFP, green fluorescent protein; nos T, nos terminator; Hyg^r, hygromycin resistance; LB, left border. (**B**) Examination of GUS expression in *GW2pro-GUS* transgenic rice. (**C**) Detection of GFP fluorescence in *GW2pro-GFP* transgenic rice. GFP imaging was performed using a LAS 4000 imager. Scale bar: 1mm.

Supplementary Table S1. The sequences of the primers used in this study.

Primer Use	Gene	Forward Primer	Reverse Primer
Gateway Cloning	*GW2*	5′-ATGGGGAACAGGATAGGGGGGAG-3′	CAACCATGCCAACCCTTGCGAGTG-3′
	GW2 Promoter	5′-GGGCTAATGGCTGTTCATTGACACTG-3′<br>5′-cagtGGATTCATGGGGAACAGGATAGGGG GGAG-3′	5′-AAGCTTGAATCCCTTCTTCTGGTCGATGTCCCTG-3′<br>5′-tcagtAAGCTTCATCAACCATGCCAACCCTTGCG-3′
	EXPLA1	5′-ATGGCCGTCTCTGTCCGTTGCTGCTTCGG-3′	5′-CTTCCACTCGTGCGTGTCGCAGGGGA-3′
Protein expression Yeast two hybrid	*GW2*	5′-tcagtGAATTCATGGGGAACAGGATAGGGGGGAG-3′	5′-tcagtGTCGACCATCAACCATGCCAACCCTTGCGA-3′
	GW2-N terminus		5′-agtcGAATTCAGTCTGGGCAGA-3′
	GW2-C terminus	5′-tagtCATATGAGCATGCGCCCT-3′	
	GW2-300 N terminus		5′-TCGTGTCGACAGCCATGTAAGAGAAG-

			3′
	EXPLA1	5′-agtcGAATTCATGGCCGTCTCTGTCCGTT-3′	5′-agtcCTCGAGCTACTTCCACTCGTGCGTG-3′
Overlapping PCR	*K237R N terminus*		5′-CACCCACCTCCCGTCGTAGCCGC-3′
	K237R C terminus	5′-GACGGGAGGTGGGTGTGGGCCGA-3′	
	K279R		5′-agtcCTCGAGCTACTCCACTCGTGCGTG-3′
Overexpression	*EXPLA1*	5′-agtcCTCGAGATGGCCGTCTCTGTCCG-3′	5′-agtcACGCGTCCCTTCCACTCGTGCGT-3′
